# Ileal Schwannoma: A Rare Cause of Pelvic Mass

**DOI:** 10.1155/2024/5572087

**Published:** 2024-02-14

**Authors:** Martin Jezovit, Hasan Bakirli, Ifrat Bakirov, Khalid Hureibi, Gultakin Bakirova, Roman Okolicany, Pavol Janac, Iveta Meciarova, Nasser Alhwaymel, Ilkin Bakirli, Augustin Prochotsky

**Affiliations:** ^1^Cyril and Methodius University Hospital, Bratislava, Slovakia; ^2^Kettering General Hospital, University Hospitals of Northamptonshire NHS Group, Kettering, UK; ^3^King Saud Medical City, Riyadh, Saudi Arabia; ^4^Al Iman General Hospital, Riyadh, Saudi Arabia; ^5^National Institute of Cardiovascular Diseases, Bratislava, Slovakia

## Abstract

The incidence of small bowel schwannomas is extremely low. In the current literature, we found just a few reported small intestine schwannomas that were located in the duodenum, jejunum, or ileum. This study reports a surprising finding of a relatively large size ileal schwannoma in a patient whose preoperative magnetic resonance imaging described a tumour in the lesser pelvis probably derived from the right adnexa. Pfannenstiel incision was made by the gynaecology team, which found a large mass lesion arising from the small intestine and occupying nearly the entire lesser pelvis. The general surgeon was invited, and pathology was successfully managed by segmental resection of the small bowel with primary end-to-end anastomosis. The histopathology study reported a submucosal tumour composed of S-100 protein-positive spindle cells, and the diagnosis of ileal schwannoma was made. The possibility of intestinal neoplasms, including schwannomas, might be contemplated in the differential diagnosis of any pelvic mass lesions. A detailed histology study and immunohistochemical stain are required for the final diagnosis of intestinal schwannomas and to rule out malignant changes, which are extremely important for the further management of patients. To the best knowledge, our case is one of the biggest intestinal schwannomas reported in the current literature.

## 1. Introduction

Schwannomas are tumours developing from Schwann cells of the sheaths of peripheral nerves, most frequently occurring in the head, neck, and extremities. In contrast, they rarely occur in the pelvis with a reported incidence of 1–3%. Pelvic schwannomas originate from the peripheral nerve sheath of the sacral nerve or hypogastric plexus [1]. In the digestive tract, they appear in less than 10% of the total number [2]. They originate from Auerbach's myenteric plexus or Meissner's submucosal plexus [3]. Previously, schwannoma and gastrointestinal stromal tumours (GIST) were often misdiagnosed as leiomyoma or leiomyosarcoma [4]. Only in recent decades, with advanced developments in immunohistochemistry, have schwannoma and GIST emerged as separate entities. The diagnosis of schwannoma is based on positive immunohistochemical staining for S-100 protein and negative results for CD117, CD34, desmin, and smooth muscle actin (SMA). In contrast, GIST is typically positive for CD117, DOG-1, and CD34 but negative for S-100 protein [5–7]. There are no specific clinical symptoms or signs of gastrointestinal (GI) schwannomas, and it depends mainly on their size and location. The main symptoms may include gastrointestinal bleeding, abdominal pain, dyspepsia, and rarely intestinal obstruction [7, 8]. Imaging by ultrasound (US) scan, contrast-enhanced computed tomography (CECT) scan, magnetic resonance imaging (MRI), 18-fluorodeoxyglucose positron emission tomography (18FDG-PET), and endoscopic studies, including endoscopic US, may reveal the tumour in the gastrointestinal tract (GIT). Due to the submucosal location of the schwannoma, an endoscopic mucosal biopsy is not conclusive. In the case of small bowel schwannomas, an endoscopic study is usually not practical. Li et al. described computed tomography (CT) criteria to differentiate gastric schwannoma from GIST [9]. However, in daily routine practice, it is challenging to distinguish between schwannoma and other tumours of GIT, especially GIST, before surgery. Intestinal schwannoma, in our case as well, was diagnosed postoperatively after a histopathologic study of the specimen. Because of the GI schwannomas' rarity, no significant data currently exists in the current literature and publishing each case may contribute great value.

## 2. Case Presentation

No written consent has been obtained from the patient as there is no patient identifiable data included in this case report. A 59-year-old female patient attended the obstetrics-gynaecology clinic with complaints of chronic lower abdominal pain for the last 2 years. She underwent a left-sided oophorectomy 33 years back for a complicated large ovarian cyst, a hysterectomy, and a right-sided oophorectomy 17 years back for symptomatic uterine fibroids. In addition, she had a history of a left-sided nephrectomy about 15 years back, for pyonephrosis. The patient was investigated as an outpatient and scheduled for surgery due to an MRI-verified tumour in the lesser pelvis. Routine blood workups were within normal values. The preoperative tumour markers were negative (Table 1).

**Figure 1:**
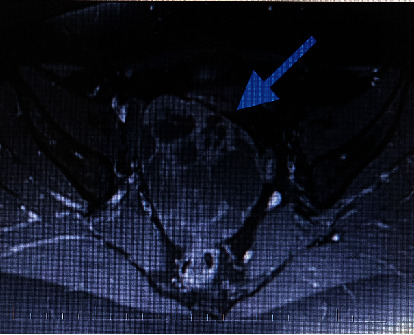
Magnetic resonance imaging, axial view: a solid-cystic tumour in the lesser pelvis with a significant solid component. The blue arrow is pointing to the pelvic tumour.

**Figure 2:**
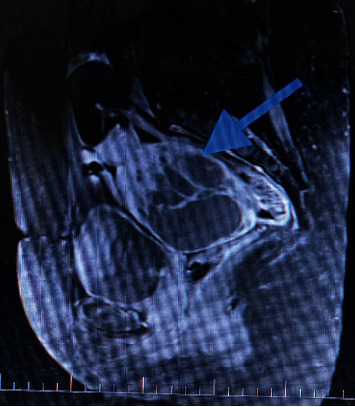
Magnetic resonance imaging, sagittal view: a solid-cystic tumour in the lesser pelvis, with thick septations. The blue arrow points to the tumour that nearly occupies the entire lesser pelvis.

ROMA (Risk of Ovarian Malignancy Algorithm) index was at a low-risk level. An MRI scan showed a solid-cystic tumour about 101 mm × 72 mm × 98 mm, probably coming out of the right adnexa, with thick septa and significantly solid components (Figures 1 and 2).

The tumour postcontrast is enhanced with diffusion restriction and no papillary projections. Mass was compressing the small bowel, sigmoid colon, rectum, and right external iliac vein. No pathologically enlarged lymph nodes nor free fluid was detected in the abdomen and pelvis.

**Figure 3:**
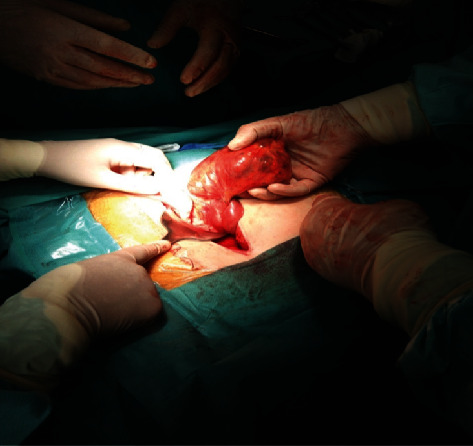
A large size intestinal schwannoma was delivered through Pfannenstiel incision.

Surgery was performed under general anaesthesia with endotracheal intubation and muscle relaxation. Initially, a cystoscopy was done, and a “double J-stent” was introduced into the right ureter by a urologist due to a history of left nephrectomy. Subsequently, a gynaecological surgery team performed a laparotomy through a Pfannenstiel (transverse suprapubic) incision. Following adhesiolysis and revision of the lesser pelvis, a large solid-cystic mass was found, which is coming out of the small bowel and stretching as far as the small pelvis. There were no findings of oncogynecological disease or remnants of organs after the hysterectomy and adnexectomy. A specialist surgeon was called to continue the surgery (Figure 3).

**Figure 4:**
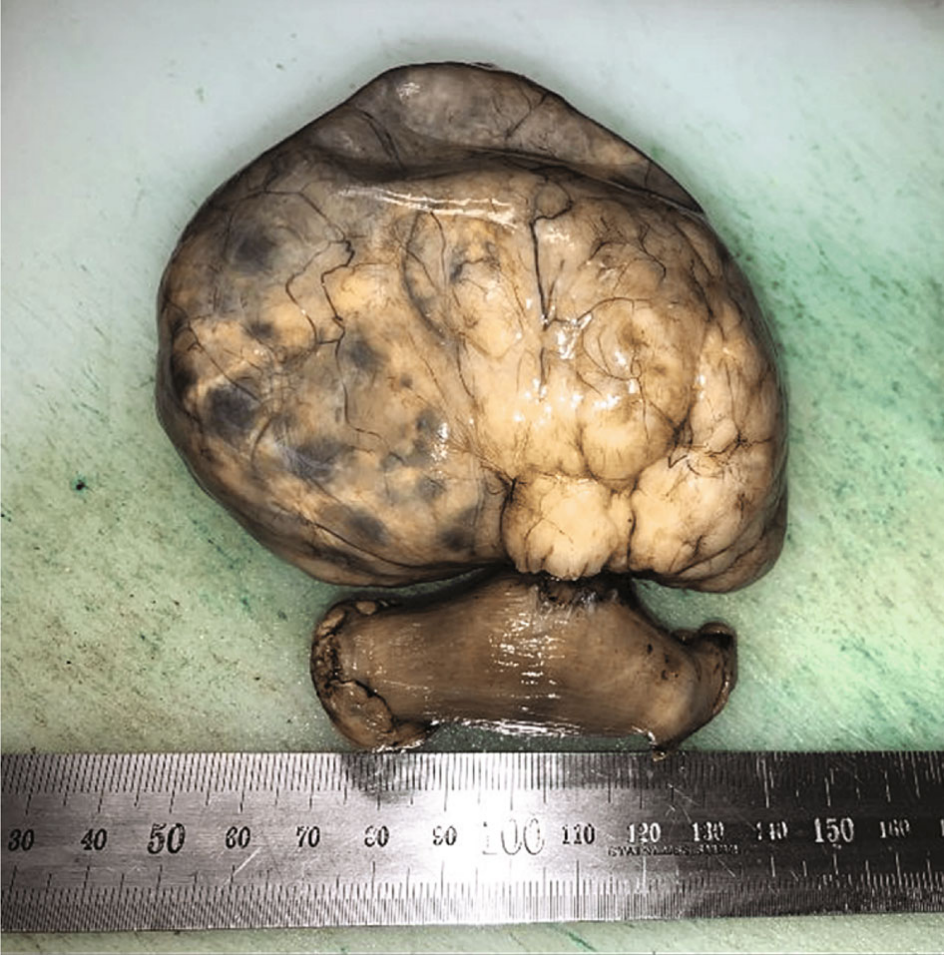
Macrospecimen contains large tumour with resected part of small bowel.

Following a revision of the organs of the abdomen and pelvis, the surgeon performed a resection of a part of the small bowel, including the tumour, which is located approximately 280 cm away from the ileocaecal valve (Figure 4).

**Figure 5:**
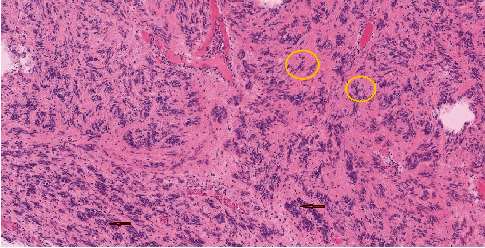
Histologic picture of ileal schwannoma showing palisade cells and Verocay bodies. Red arrows are showing the palisade cells, and yellow circles are marking the adjacent Verocay bodies.

Then, the gastrointestinal passage was renewed through handmade enteroenteral “end-to-end” anastomosis in two layers. Following a hemostasis and revision, a Tygon drain tube was introduced into the Douglas pouch. The abdominal cavity was closed by each anatomic layer. Operation time was 67 minutes, and estimated blood loss was about 90 ml. Postoperatively, the patient had haematuria, which was successfully managed conservatively by the urology team. The abdominal drain during the first two days was discharging serosanguinous fluid and was removed on the 3 d postoperative day, her clinical condition and haematuria gradually improved, and the passage of food through the digestive tract was restored without difficulties. Prior to her discharge, the “double J-stent” had been extracted by a urologist, with no subsequent haematuria and with favourable renal function tests. The patient was discharged in good condition on the 6^th^ postoperative day. Histopathology showed relatively well-defined tumour arising from a submucous layer of small bowel with variable composition, reticular/microcystic arrangement predominates, and myxoid parts; irregular cellular spindle cell appearance of the Antony A area, palisade, “Verocay bodies,” focally accentuated vascularisation in the reticular parts in the form of irregular, often dilated, septated blood vessels with hyalinosis of the wall, extravasation of erythrocytes, and perivascular deposits of hemosiderin; intercellular spaces with hyalinosis, often pseudocystic spaces; and peripheral noncontinuous lymphocytic border with the formation of lymphoid aggregates (Figure 5).

Immunohistochemically, S-100 +++, VIM + and GFAP, EMA, OSCAR, CD34, CD117, HMB45, MelA, DEZ, CD10, CD99, FXIIIa, ChromA, desmin, synaptophysin, ER, and inhibitin all were negative. Ki67 was less than 1%. The report concluded that histopathologic study and immunohistochemical staining are consistent with an intestinal schwannoma—an ileal submucosal neurilemmoma (a rare tumour in the small bowel, developing in the GIT from Auerbach's or Meissner's plexus). No signs of malignancy were detected. Completeness of resection was confirmed by histology study.

The postoperative period after discharge to home was uneventful, and laparotomy wound is healed by primary intention. Within 4 years of follow-up time, the patient remained asymptomatic.

## 3. Discussion

Schwannomas were first referred to in 1910 by Verocay and characterised in more detail in 1935 by Stout [10]. In 1988, Daimaru et al. suggested a concept of a benign intestinal schwannoma and thoroughly documented its morphological and phenotype characteristics [4]. An intestinal schwannoma belongs to the class of gastrointestinal mesenchymal tumours (GIMTs) [11]. In clinical practice, for differential diagnostics of schwannomas within the group of mesenchymal tumours, it is necessary to perform an immunohistochemical examination, which is, technically speaking, most often not possible to be done in the small bowel region until resecting the segment containing the tumour to undergo histological evaluation. Since the findings of small bowel schwannomas are extremely rare, their incidence in the population is unclear and has only been estimated in some existing studies [2, 7]. In the current digital literature, we found that only a few small bowel schwannomas have been described [2, 7, 12–22] that localised to the duodenum, jejunum, or ileum. The majority of patients (about 2/3) were females. The mean age was 58.86 years, and the median age was 66 years. Our case report describes the discovery of a relatively large schwannoma in a patient under a gynaecologist's care, the original presumption being, based on the medical history of previous surgical interventions by the gynaecology team and the localisation of the tumour in the lesser pelvis region. So it was preoperatively presumed that the tumour had originated from the adnexal remnants. Due to that, the patient had been operated on at a gynaecology theatre, and a transverse suprapubic Pfannenstiel incision was used for the laparotomy. This fact, subsequently, made it difficult for the surgeon assigned to revise the larger area of the peritoneal cavity and the surgical performance itself. The resection of a part of the ileum, including the tumour, was performed while adhering to oncological principles, as during the procedure, the nature of the tumour was unknown. No postoperative complications relating to the resection and anastomosis of the intestine are observed. It should be noted that originally, the patient's attitude to tolerate pain and some discomfort was due to her multiple previous surgeries, and the patient was accepting them as a normal postoperative condition. This fact probably contributed to the tumour acquiring a relatively large size (101 mm × 72 mm × 98 mm). In comparison, the average maximum diameter of all published small bowel schwannomas (including duodenal) is 51.07 mm. Our patient had follow-ups within 4 years in the surgical outpatient clinic every 6 months and remains symptom-free. Literature data also supports the idea that GI schwannoma is a benign condition and nearly no malignant transformation is observed [2, 7, 12–15], and the long-term follow-ups of patients with schwannomas of the gastrointestinal tract have not shown any propensity to recurrence following complete excision [2].

## 4. Conclusions

Small bowel schwannoma is one of the rare tumours involving the gastrointestinal tract. It is a benign pathology with female predominance. Any pelvic mass differential diagnosis should consider bowel tumours, including intestinal schwannomas. Surgical resection is the curative treatment. Histology study and immunohistochemical stains are mandatory to diagnose intestinal schwannoma and rule out malignant changes, which are extremely important for the further management of patients. To the best knowledge, our case is one of the biggest intestinal schwannomas reported in the current literature. Because of the rarity of pathology, publishing each intestinal schwannoma case may contribute to statistical and scientific interest.

## Figures and Tables

**Table 1:** Tumour markers.

Tumour markers	Result	Normal value	Unit
CEA	0.95	0-3	ng/ml
CA 19-9	10.10	0-37	U/ml
CA 125	13.5	0-35	U/ml
Human HE4	55.60	0-83	pmol/l

CEA: carcinoembrionic antigen; CA: cancer antigen; HE4: human epididymis protein 4.

## Data Availability

The data supporting the results of this study can be provided by corresponding author if requested by the editors of the journal.
